# Resveratrol Alleviates Dextran Sulfate Sodium-Induced Acute Ulcerative Colitis in Mice by Mediating PI3K/Akt/VEGFA Pathway

**DOI:** 10.3389/fphar.2021.693982

**Published:** 2021-08-23

**Authors:** Fang Zhu, Jujia Zheng, Fang Xu, Yiyuan Xi, Jun Chen, Xiangwei Xu

**Affiliations:** ^1^ Department of Gastroenterology, The First People’s Hospital of Yongkang Affiliated to Hangzhou Medical College, Jinhua, China; ^2^ School of Pharmaceutical Sciences, Wenzhou Medical University, Wenzhou, China; ^3^ Department of Pharmacy, The First People’s Hospital of Yongkang Affiliated to Hangzhou Medical College, Jinhua, China

**Keywords:** resveratrol, ulcerative colitis, phosphoinositide 3-kinase, protein kinase B, vascular endothelial growth factor A

## Abstract

Ulcerative colitis (UC) is a chronic inflammatory disease that affects the colon, and its incidence is on the rise worldwide. Resveratrol (RSV), a polyphenolic compound, was recently indicated to exert anti-inflammatory effects on UC. Consequently, the current study was conducted to investigate the mechanism of RSV on alleviating UC in mice by mediating intestinal microflora homeostasis. First, potential targets that RSV may regulate UC were screened using the TCMSP database. Next, mice were treated differently, specifically subjected to sham-operation and dextran sulfate sodium (DSS) induction, and then treated or untreated with RSV. Disease Activity Index (DAI) and Hematoxylin-Eosin (HE) staining were employed to analyze the pathological changes of mice colon. In addition, the expression patterns of inflammatory factors in spleen tissues were detected using ELISA, while the protein expression patterns of phosphoinositide 3-kinase (PI3K), protein kinase B (Akt), and vascular endothelial growth factor A (VEGFA) in colon tissues were determined by means of immunohistochemistry (IHC) and Western blot analysis. Moreover, changes in intestinal flora and metabolite diversity in UC were analyzed by metabonomics. It was found that RSV played inhibitory roles in the PI3K/Akt pathway in mice. Meanwhile, the administration of RSV induced downregulated the expressions of TNF-α, IFN-γ, IL-1β, IL-6, and IL-4. The six floras of *Haemophilus* and *Veillonella* were significantly enriched in UC, while *Clostridium*, *Roseburia*, *Akkermansia*, and *Parabacteroides* were found to be enriched in control samples. Lastly, it was noted that *Akkermansia* could regulate the intestinal flora structure of UC mice through triacylglycerol biosynthesis, glycerol phosphate shuttle, cardiolipin biosynthesis, and other metabolic pathways to improve UC in mice. Altogether, our findings indicate that RSV suppressed the activation of the PI3K/Akt pathway and reduced the VEGFA gene expression to alleviate UC in mice.

## Introduction

Ulcerative colitis (UC) is one of the most common forms of inflammatory bowel disease, characterized by damage to colonic epithelial cells (Kuwada et al., 2021). The pathogenesis of UC is attributed to various factors, ranging from hereditary susceptibility to epithelial barrier defects, dysimmune response, and environmental factors (Ungaro et al., 2017). Clinically, UC is mostly diagnosed in late adolescence or early adulthood, but the disease can manifest at any age, with typical symptoms including bloody diarrhea (da Silva et al., 2014). Currently, total proctocolectomy and ileal pouch-anal anastomosis are regarded as the gold standard for surgical treatment of refractory UC (Scoglio et al., 2014). Although there are numerous drug treatment options available for UC, its nonresponsive behavior to systemic steroid therapy remains a huge challenge in the field of inflammatory bowel disease (Hoffmann et al., 2019).

Rhizoma smilacis glabrae (RSG), a widely used traditional Chinese medicine, is known for its ability to deoxidize, dehumidify, and relieve joint movement, while its chemical composition is known to include resveratrol (RSV) (Liang et al., 2019). RSV is a polyphenolic compound found in grapes, red wine, chocolate, and some berries and roots, which possesses antioxidant, anti-inflammatory, and anticancer properties (Semba et al., 2014). Moreover, RSV has been shown to be efficacious against UC; for example, supplementation with RSV improves disease activity and quality of life in patients with UC, at least in part, by reducing oxidative stress (Samsamikor et al., 2016). Besides, RSV can directly regulate the phosphoinositide 3-kinase (PI3K)/protein kinase B (Akt) pathway, such that RSV reduced intestinal inflammation after irradiation by regulating the PI3K/Akt pathway (Radwan and Karam, 2020). Meanwhile, the PI3K/Akt pathway is an intracellular pathway, which plays an indispensable role in the control of the cell cycle (Ghafouri-Fard et al., 2021). Furthermore, the PI3K/Akt pathway is implicated in the regulation and release of proinflammatory cytokines, which in turn participates in the development of UC, while PI3K/Akt expressions are augmented in mice with UC (Huang et al., 2011). On the other hand, vascular endothelial growth factor A (VEGFA) has previously been identified as a central target gene of RSV and has been known to exhibit better affinity with RSV (Wang W. et al., 2020). In addition, VEGFA can influence physiology and tumor-induced angiogenesis, whereas elevated VEGFA gene expressions are associated with tumor progression, recurrence, and survival (Andreozzi et al., 2014). More strikingly, UC patients are known to exhibit higher levels of VEGFA than normal controls (Pousa et al., 2011). Consequently, the current study aimed to verify how RSV regulates the PI3K/Akt/VEGFA pathway to relieve UC.

## Materials and Methods

### Ethics Statement

All experimental procedures are proceeded in accordance with the instructions of the Experimental Animal Care and Use Ethics Committee of The First People's Hospital of Yongkang Affiliated to Hangzhou Medical College and Wenzhou Medical University. Extensive measures were undertaken to minimize the suffering of the experimental animals.

### Network Pharmacological Analysis

Firstly, the compound components of RSG were retrieved through the TCMSP database (http://tcmspw.com/tcmsp.php), and condition screening was performed and set as oral bioavailability (OB) > 10% and drug-likeness (DL) > 0.1, which reared a total of 34 compound components. Next, the multitargets corresponding to the compounds in RSG were searched using the TCMSP database. Meanwhile, genes related to UC were identified through the GeneCards database (https://www.genecards.org/), and Jvenn (http://jvenn.toulouse.inra.fr/app/example.html) was utilized to search for the overlap of compound targets and UC targets. To further conduct enrichment analysis on the overlap between drug targets and disease targets, WEB-based GEne SeT AnaLysis Toolkit (http://www.webgestalt.org/) was adopted to find targets (the species condition was restricted to “*Mus musculus*” and the database was selected to be “KEGG”). In addition, to find the core targets involved in the pathway, the STRING website (https://string-db.org/) was employed to analyze the gene association interaction (minimum required interaction score = 0.4), and the Cytoscape 3.5.1 software was used to visualize the association interaction network relations. Finally, the Cytoscape 3.5.1 software was employed to construct the drug-compound-target direct relationship network. The flowchart of the current study is shown in Supplementary Figure 1.

### Study Subjects

Resveratrol (RSV, Cat. NEL-1291) was procured from NeoCell (Irvine, California, United States). Meanwhile, a total of 80 male BALB/c mice (aged 6–8 weeks; calculated mean weight of 18.49–23.72 g) were purchased from the Animal Experimental Center of Guangzhou University of Chinese Medicine, Guangzhou, China. The experiment was commenced after one week of adaptive feeding. The mice were randomly divided into the following four groups (*n* = 20): the normal control group (NG group), the model control group (MG group), the RSG group (RG group), and the positive control group (PG group). Mice in the MG group were treated with free drinking of 5% dextran sulfate sodium (DSS, MW: 5,000) for 7 days, and then this changed to free access to distilled water for 7 days. Meanwhile, the mice in the NG and MG groups received the same amounts of distilled water. On the other hand, mice in the RSG group were treated with RSV at a dosage of 100  mg/kg.d. Sulfasalazine (SASP; Jiuzhou Pharma, Zhejiang, China; CAS No: 599-79-1), as a positive control drug, was administered to mice in the PG group at a dosage of 200  mg/kg.d. RSV and SASP treatments were performed every day from day 1 to day 7. At the beginning of modeling, mice were given gavage every day at the same time and continued to intervene until the end of the experiment. During the experiment, the body weight, fecal consistency, and blood in the stool of the mice were all monitored every day, and “DAI (Disease Activity Index, Table 1) score” was used to describe and indicate the “severity of UC.” After the experiment, the animals were euthanatized and samples were collected for subsequent analyses. Fresh fecal samples were also collected using metabolic cages and immediately stored at −80°C for later use. The colon contents were stored at −80°C for intestinal microflora analyses.

**TABLE 1:** The clinical disease activity index (DAI) score.

Score	Weight loss (%)	Stool consistency	Rectal bleeding
0	None	Normal	Normal
1	1–5		±
2	5–10	Loose stool	+
3	10–15		++
4	More than 15	Watery diarrhea	+++

DAI =  (weight loss + stool consistency + rectal bleeding)/3.

### Hematoxylin-Eosin Staining

The distal section of the colon was excised, and the rest was fixed in 4% paraformaldehyde and embedded in paraffin. Next, 5 μm thick sections were stained with Hematoxylin-Eosin (HE) and observed under a BH22 optical microscope (Olympus, Tokyo, Japan) at 100x magnification. The tissue sections were observed under an optical microscope and scored according to the 5-score grading system reported by Xiong et al. as follows (Abdin, 2013): a score of 0 indicated no inflammation; a score of 1 indicated low leukocyte infiltration and no structural changes; a score of 2 indicated moderate leukocyte infiltration, dilation, and malformation, with no ulcers in the mucosal gland duct; a score of 3 indicated severe leukocyte infiltration, accompanied by increased blood vessel density and thickening of the intestinal wall; a score of 4 indicated a large amount of white blood cell infiltration, high blood vessel density, enlarged and deformed glands, obvious loss of goblet cells, thickened intestinal wall, and complicated ulcers.

### Enzyme-Linked Immunosorbent Assay

Colonic tissues were homogenized in phosphate-buffered saline (PBS) buffer (pH 7.4) and centrifuged at 14,000 g at 4°C for 15 min. The supernatant was then collected and subjected to BCA quantification. Based on the quantitative results, the same amount of protein was selected from each experimental group selected and the levels of cytokines (TNF-α, IFN-γ, IL-1β, IL-4, IL-6, and IL-10; pg/ml/mg) were quantified using ELISA kits (Pierce Biotechnology, Rockford, IL) according to the manufacturer’s protocol.

### Immunohistochemistry

After the experiment, the colon tissues of mice were collected, and the paraffin-embedded mouse colon tissues (thickness of 4 µm) were fixed with formalin. Following deparaffinization and hydration, the sections were treated with 3% hydrogen peroxide to block endogenous peroxidase activity, and antigenic repair was achieved by boiling the sections in citrate buffer for 30 min. Next, the sections were sealed with 5% bovine serum albumin (BSA) and incubated overnight at 4°C with primary antibodies PCNA (AB29, dilution ratio of 1: 10,000, Abcam, Cambridge, United Kingdom), VEGFA (AB133352, dilution ratio of 1: 250, Abcam), PI3K (95,625, dilution ratio of 1: 400, CST, Boston, Massachusetts), and Akt (95,625, 1: 400, CST). Afterward, the sections were stained with diaminobenzidine (DAB, Boster Biological Technology, Pleasanton, CA) substrate, and the nuclei were stained with hematoxylin.

### *In Vitro* Culture

The frozen-thawed colon tissues were broken into fragments measuring around 4 × 2 × 1 mm^3^. Next, one to three segments from each biopsy were selected as day 0 (D0) controls and fixed for histological evaluation. The remaining fragments were treated with DSS, DMSO, 740Y-P (activator of PI3K/Akt), or RSV. The fragments were then individually placed into four-well cell culture plates (Thermo Fisher, Waltham, MA) containing 300 μL medium (McCoy’s 5a medium) with bicarbonate added with 25 mM HEPEs (Gibco Life Technologies Corp., Grand Island, NY), 0.1% HSA, 3 mM glutamine, 30 μg/ml penicillin, 50 μg/ml streptomycin, 2.5 μg/ml transferrin (Thermo Fisher), 4 ng/ml selenium (Sigma-Aldrich, St Louis, MO, United States), and 50 μg/ml ascorbic acid (Sigma-Aldrich). Later, 150 μg/ml 740Y-P (Bio-Techne, Cambridge, United Kingdom) and 1% of DMSO (Sigma-Aldrich) were added for the first 48 and 24 h, respectively, at 37°C, in humidified air with 5% CO_2_. On day 1, the medium was renewed with fresh medium supplemented with 10 ng/ml insulin (Sigma-Aldrich) and 1 ng/ml HFSH (Gonal F, Merck Serono, United Kingdom), and the tissues were cultured for 5 days, with half of the medium being renewed every other day. After 0, 1, 3, and 6 days of culture, a total of 56 cortical strips were fixed in 4% paraformaldehyde for 24 h for histological and immunohistological analysis. Overall, 21 fragments cultured for 0, 1, or 6 days were used for Western blot analysis, and colon tissues isolated from 35 fragments were used for gene expression analysis with real-time quantitative polymerase chain reaction (RT-qPCR).

### RT-qPCR

Colon tissues from different groups were collected and lyzed using TRIzol kits (Thermo Fisher, 10296010) to extract the total RNA content from the tissue samples. The complementary (cDNA) Synthesis kits (HiScript First Strand cDNA Synthesis Kit, Vazyme Biotech, China, R111-01) were then used to synthesize cDNA with a 20 μl system. The reaction conditions were as follows: 37°C, 15 min; 85°C, 5 s. β-Actin was used as the internal reference to analyze mRNA expression. The 2^−ΔΔCT^ method was applied to the measurement of the target gene relative expression levels (Supplementary Table 1).

### Western Blot Analysis

Colon and spleen tissues of mice were collected, and the protein content was extracted using radioimmunoprecipitation assay (RIPA) lysate. The concentration of the obtained protein was determined with bicinchoninic acid kits (BCA). A total of 50 μg of protein samples was then isolated with sodium dodecyl sulfate-polyacrylamide gel electrophoresis (SDS-PAGE). The isolated proteins were transferred onto a polyvinylidene fluoride membrane and sealed with 5% skimmed milk at room temperature for 2 h. Next, the tissues were incubated with the primary antibody overnight at 4°C. The main incubation antibodies included anti-VEGFA (ab52917, dilution ratio of 1 : 10,000, Abcam), anti-Akt (ab38449, dilution ratio of 1 : 1,000, Abcam), anti-PI3K (ab140307, dilution ratio of 1 : 2000, Abcam), and anti-β-actin  (ab179467, dilution ratio of 1 : 5,000, Abcam). Afterward, the cultivated tissues were further incubated with goat anti-rabbit immunoglobulin G (IgG) for 1 h at room temperature. The membrane was then incubated with ECL working solution (EMD Millipore, Bedford, Massachusetts, United States) for 1 min at room temperature. The excess ECL reagent was removed, the membrane was sealed, and an X-ray film was placed in the carton for 5–10 min of exposure, followed by development and fixing. The grayscale of each band in Western blot images was quantified using the ImageJ analysis software. β-Actin was used as the internal reference. The experiment was repeated three times to obtain the mean value.

### Metabolomics Analysis

The intestinal flora database GMrepo (https://gmrepo.humangut.info/), gutMEGA (http://gutmega.omicsbio.info/, condition: ulcerative colitis/normal, *p* value < 0.05), and gutMDisorder (http://bio-annotation.cn/gutMDisorder) were retrieved to uncover the gut bacteria related to UC, with Jvenn being used to obtain the intersection.

The relevant samples of UC were searched through the Metabolomics Workbench database (https://www.metabolomicsworkbench.org/), downloading the data matrix (Alterations in Lipid, Amino Acid, and Energy Metabolism Distinguish Crohn Disease from Ulcerative Colitis and Control Subjects by Serum Metabolomic Profiling Reversed phase POSITIVE ION MODE). The sample was comprised of 20 control and 20 UC samples. The R language “limma” package (http://www.bioconductor.org/packages/release/bioc/html/limma.html) was used to perform differential analysis of the metabolite expression profiles, with |logFC|>0.6 and *p* < 0.05 as the screening criteria for differential metabolites. The R language “pheatmap” package (https://cran.r-project.org/web/packages/pheatmap/index.html) was applied to plot a heat map of the differential metabolite expression. The MetaboAnalyst tool (https://www.metaboanalyst.ca/) was adopted for enrichment analysis of differential metabolites. Using the VMH database (https://www.vmh.life/?tdsourcetag=s_pctim_aiomsg), the microbes with differential metabolites were predicted, and Jvenn was adopted to intersect the differential microbes of UC to predict the key to UC microorganisms. The distribution and box plot of the relative abundance of key microorganisms in both healthy and UC samples were further searched using the GMrepo database.

### Statistical Analysis

Statistical analyses were performed using the SPSS 21.0 version (IBM, Armonk, New York). Measurement data were expressed by mean ± SD. Paired *t*-test was adopted for comparisons of the paired data between two groups with normal distribution and homogeneity of variance, while unpaired *t*-test was performed for comparisons of unpaired data. One-way analysis of variance (ANOVA) or repeated measures ANOVA was conducted for multiple group comparison, followed by Tukey’s post hoc test. The Kaplan–Meier method was used to calculate the survival rate, and log-rank test was used for univariate analysis. Pearson’s correlation was used to analyze the correlation of the observed indexes. A value of *p* < 0.05 was considered statistically significant.

## Results

### RSV Contributes to UC Development

**FIGURE 1:**
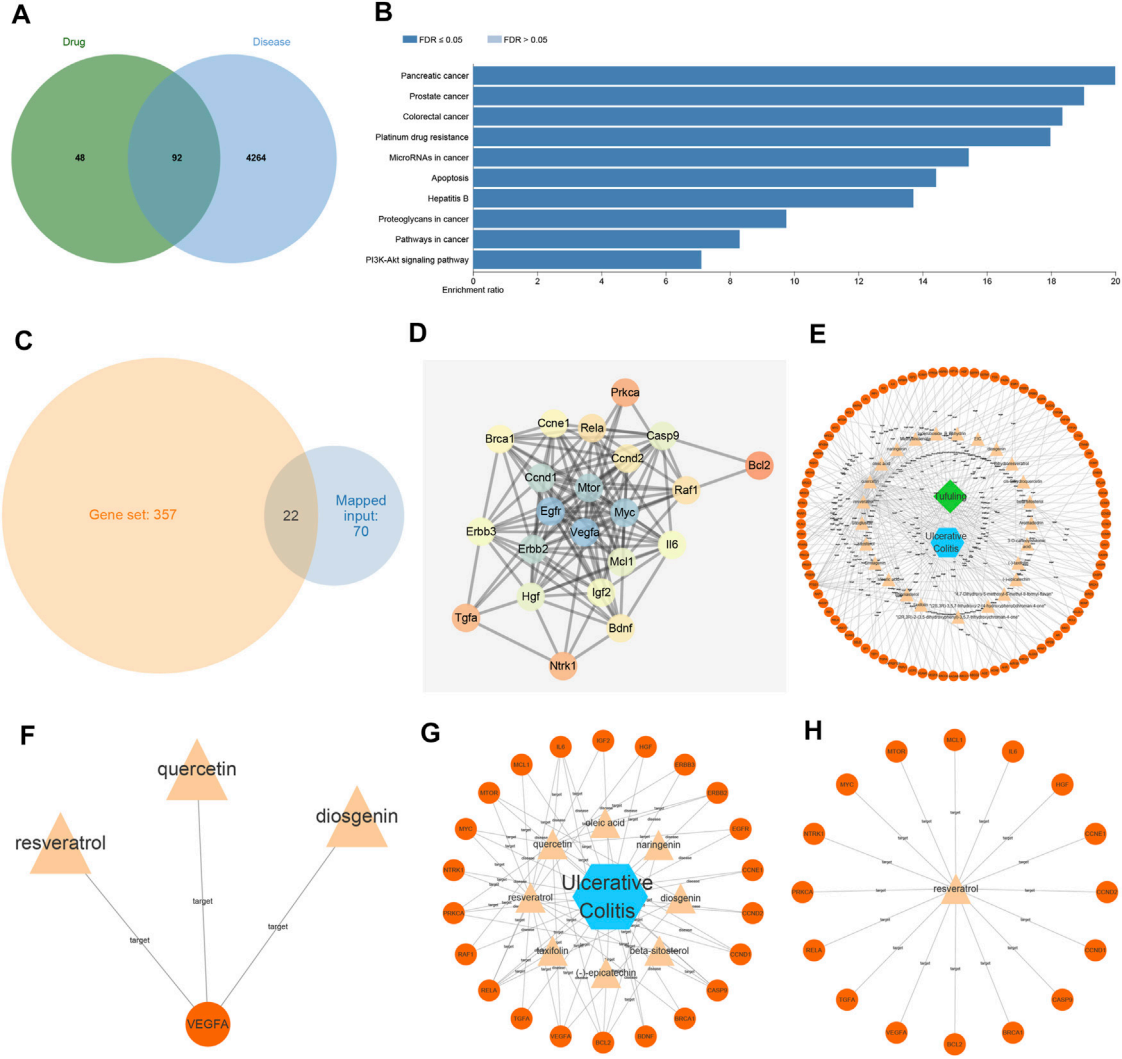
Resveratrol (RSV) functions as a potential target for the therapy of ulcerative colitis (UC). **(A)** The overlapping genes between compound targets and UC targets in 92 RSG examined by Jvenn. **(B)** Analysis of KEGG enrichment of overlapping genes between drug targets and disease targets. **(C)** The overlapping zone between genes involved in enrichment analysis and genes involved in PI3K/Akt pathway. **(D)** The correlation and interaction analysis network between genes involved in the PI3K/Akt pathway; the color scale of the circle from orange to blue indicated that the degree value was from small to large. **(E)** Disease-compound-target relationship network diagram; orange represented targets, yellow compound, blue disease, and green traditional Chinese medicine. **(F)** Compounds that regulated core targets. **(G)** Compounds that controlled genes in the PI3K/Akt pathway. **(H)** Target genes regulated by RSV. VEGFA, vascular endothelial growth factor A.

RSG is widely used in traditional Chinese medicine to deoxidize, dehumidify, and relieve joint movement. Moreover, the chemical composition of RSG has been systematically confirmed, wherein some compounds are known to exert effects on antioxidation, anti-inflammatory, immune regulation, urinary lowering, and liver protection (Liang et al., 2019). Initially, we searched the compound components in RSG using the TCMSP database, retrieved a total of 34 compound components, and then searched for the corresponding targets of compounds in *Poria cocos* through the TCMSP database. In addition, genes related to UC were retrieved through the GeneCards database, and Jvenn was performed to find the intersection of the 92 overlapping genes of compound targets and UC targets in RSG (Figure 1A). KEGG enrichment analysis was further performed on the overlapping genes of drug targets and disease targets, and the pathways in which the genes were primarily involved were obtained (Figure 1B), including various cancer pathways, apoptosis pathways, and PI3K/Akt pathways. Among them, various studies have highlighted that inhibiting the PI3K/Akt pathway can improve UC (Chen et al., 2017). Furthermore, the results of enrichment analysis showed that 22 genes participated in the PI3K/Akt pathway (Figure 1C).

Aiming to elucidate the core targets participating in the PI3K/Akt pathway, we obtained the correlation and interaction analysis network of 22 genes using the STRING website (Figure 1D). It was found that VEGFA was at the core of the interaction network (degree = 19). Moreover, the relationship network diagram of disease-compound-target was obtained through the Cytoscape software (Figure 1E), and further analysis of VEGFA-related node revealed that three compounds (quercetin, RSV, and diosgenin) acted on the target gene VEGFA (Figure 1F). The genes involved in PI3K/Akt pathway were further analyzed with the Cytoscape software and the genes in the PI3K/Akt pathway primarily acted on eight compounds (Figure 1G), among which RSV regulated the most target genes, reaching 16 target genes (Figure 1H). Altogether, these findings indicated that RSV may regulate VEGFA through the PI3K/Akt pathway, thus affecting UC.

### RSV Attenuates DSS-Induced UC in Mice

**FIGURE 2:**
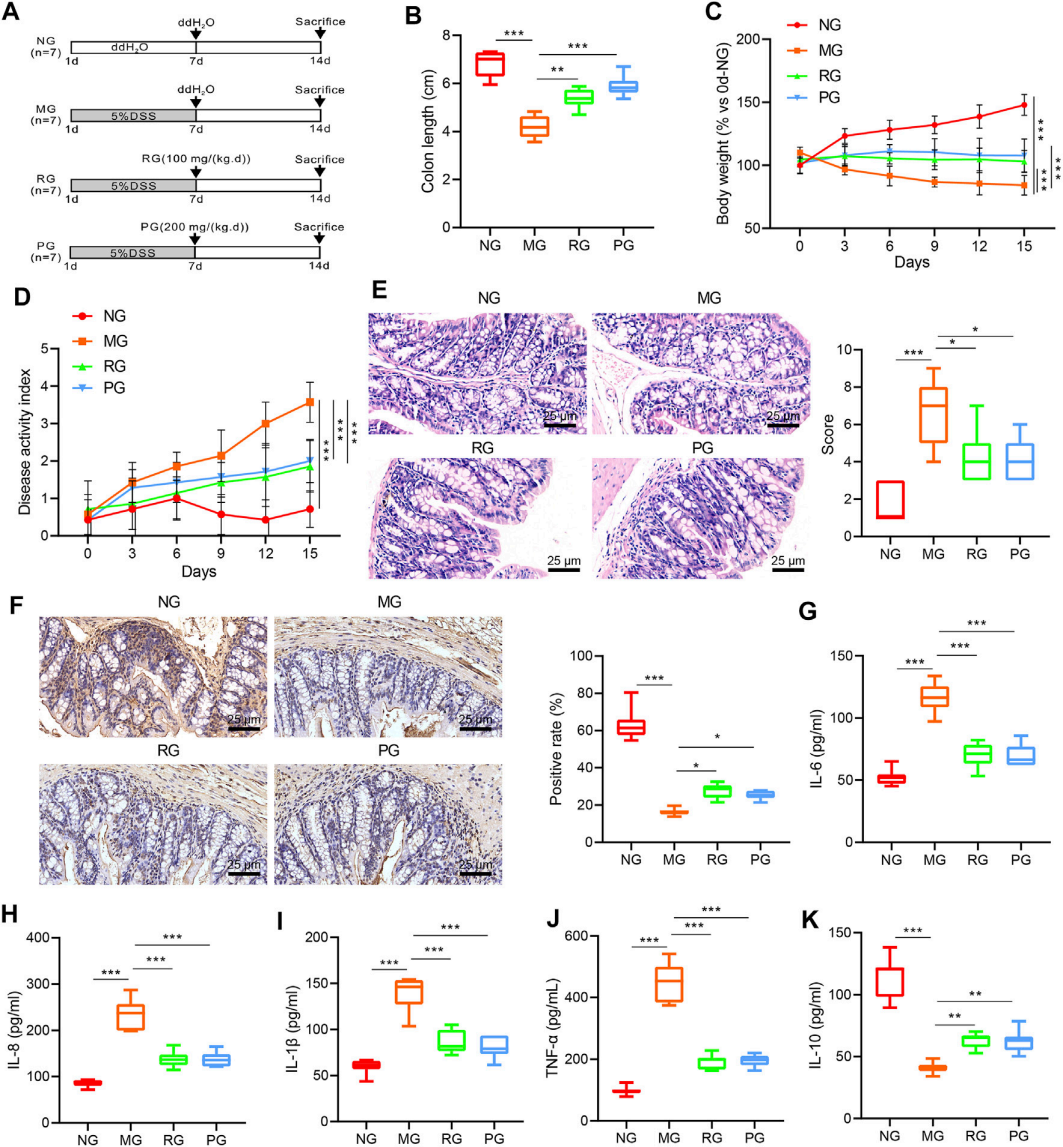
RSV can bring positive results to UC. **(A)** Schematic diagram of animal experiment. **(B)** The colon length in MG, NG, RG, and PG mice was compared. **(C)** The curve of weight change of mice in different treatment groups was analyzed. **(D)** DAI disease scores of mice in different treatment groups were evaluated. **(E)** The pathological sections of the colon of mice in different treatment groups were observed using HE staining (100 ×, scale bar = 100 µm). **(F)** PCNA expression in the colon of mice in different treatment groups was measured using IHC (100 ×, scale bar = 100 µm). **(G–K)** The expression of IL-6, IL-8, IL-1β, TNF-α, and IL-10 in the colon tissues of mice in different treatment groups was monitored using ELISA. *n* = 20. Measurement data were expressed by mean ± SD. One-way ANOVA or repeated measures ANOVA was conducted for multiple group comparison, followed by Tukey’s post hoc test. ^*^ *p* < 0.05, ^**^ *p* < 0.01, and ^***^ *p* < 0.001. NG, normal control group; MG, model control group; RG, resveratrol group; PG, positive control group.

To further investigate the specific effects of RSV on UC, we used DSS to establish mouse models of UC. The schematic diagram of the experimental process is shown in Figure 2A. In comparison with NG mice, the colon length of mice in the MG group was observed to be markedly shorter (*p* < 0.01). Meanwhile, the administration of RSV and SASP induced an evident increase in colon length (*p* < 0.05) (Figure 2B). The change in body weight curve illustrated that the body weight of mice in the NG group increased with age, while the body weight of mice in the MG, RG, and PG groups all exhibited a decrease at the same ages. Following treatment with RSV and SASP, the body weight of mice in the RG and PG groups was increased slowly and then stabilized compared with the MG group (Figure 2C). In addition, the DAI score index showed that mice in the MG group showed early blood in the stool, and blood was apparent from the “DAI” curve, while the blood in the stool of mice of the same age was notably reduced compared with that of the RG group (Figure 2D). Furthermore, HE staining demonstrated that the colon of mice in the MG group was severely damaged compared to those in the NG group, while no significant structural disorders were observed in the RG and PG groups (Figure 2E). PCNA was further applied to assess the proliferation level of intestinal epithelial cells (Sharma et al., 2020), and PCNA of the MG group was found to be lower than that of the NG group, while the PCNA in the colon of the RG group and the PG group was higher (Figure 2F).

The effect of RSV on the expressions of inflammatory factors in the colon tissue of mice with UC was further analyzed with the help of ELISA. It was found that the levels of proinflammatory cytokines IL-6, IL-8, IL-1β, and TNF-α in colon of mice in the MG group were all significantly elevated (*p* < 0.01), while the expression levels of anti-inflammatory cytokine 1 L-10 were markedly declined in comparison with those in the NG group (*p* < 0.05). On the other hand, compared with the MG group, the expression levels of IL-6, IL-8, IL-1β, and TNF-α in the colon of the RG and PG groups were all significantly reduced (*p* < 0.01), whereas the expression of 1 L-10 was notably increased (*p* < 0.01) (Figures 2G–K). Taken together, these findings suggested that RSV intervention can reduce the expression of inflammatory factors in mice with UC, thus attenuating the symptoms of UC.

### RSV Blocks PI3K/Akt Pathway to Reduce VEGFA Expression

**FIGURE 3:**
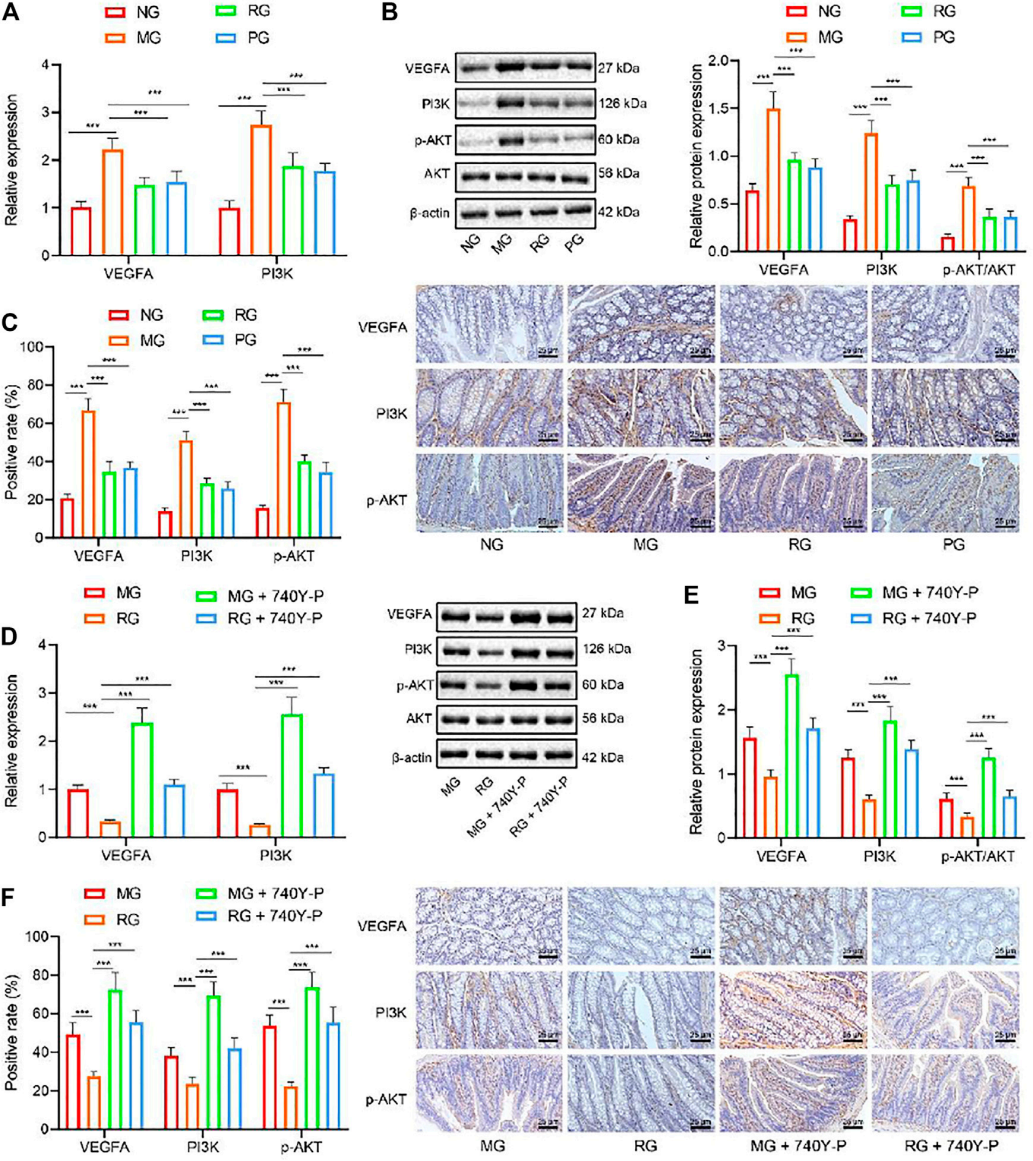
RSV inhibits the PI3K/Akt pathway activation and reduces the VEGFA gene expression. **(A)** The expression of VEGFA and PI3K and genes in colon tissues was analyzed by RT-qPCR. **(B)** The expression of VEGFA, PI3K, p-Akt, and Akt protein in colon tissues was detected by Western blot analysis. **(C)** Expression of VEGFA and Akt protein in colon tissues was analyzed by IHC. **(D)** The expression of VEGFA and PI3K in colon tissues after the addition of PI3K/Akt activator was determined with RT-qPCR. **(E)** Western blot analysis of the expression of VEGFA, PI3K, and p-Akt/Akt ratio was in colon tissues after the addition of PI3K/Akt activator. **(F)** The expression of VEGFA and p-Akt protein in colon tissue after adding PI3K/Akt activator was determined with IHC. ^#^ *p* < 0.05 vs*.* VEGFA, PI3K, and Akt expression in MG. ^##^ *p* < 0.01. *n* = 20. Measurement data were expressed by mean ± SD. One-way ANOVA was conducted for multiple group comparison, followed by Tukey’s post hoc test. NG, normal control group; MG, model control group; RG, resveratrol group; PG, positive control group; VEGFA, vascular endothelial growth factor A; 740Y-P, PI3K/Akt activator.

The results of network pharmacology analysis suggested that RSV may reduce the expression of VEGFA through the PI3K/Akt pathway to exert functions on UC. To verify the aforementioned, we analyzed the expression patterns of PI3K/Akt pathway-related genes with the help of RT-qPCR. It was found that, compared with that in the NG, the VEGFA, PI3K, and Akt gene expression levels in the colon tissues of the MG were all significantly enhanced (*p* < 0.01). Meanwhile, compared with the MG group, the VEGFA and PI3K gene expression levels in the colon tissues of the RG were all significantly reduced (*p* < 0.05) (Figure 3A). In addition, the results of Western blot analysis and IHC illustrated that mice in the MG group presented with markedly elevated VEGFA, PI3K, p-Akt, and Akt protein expressions when compared to those in the NG (*p* < 0.01). Opposingly, when compared to that in MG, the protein expressions of VEGFA, PI3K, and Akt in RG were decreased (*p* < 0.05) (Figures 3B,C).

To further define the role of RSV in the PI3K/Akt pathway by regulating VEGFA expression, we introduced a PI3K/Akt activator (740Y-P) in mice and adopted RT-qPCR, Western blot analysis, and IHC, which revealed that the protein expression levels of VEGFA, PI3K p-Akt, and Akt were all significantly reduced in the colon tissues in the RG group while being increased in the colon tissues in the MG + PI3K/Akt activator group compared with the RG group (Figures 3D–F). The above results indicate that PI3K/Akt activator can reverse the reduction of RSV on VEGFA, PI3K, and Akt expression. Altogether, these findings indicated that RSV may be used to treat UC by inhibiting PI3K/Akt pathway to play anti-inflammatory and other active roles.

### Inhibition of PI3K/Akt Pathway Reduces DSS-Induced UC in Mice

**FIGURE 4:**
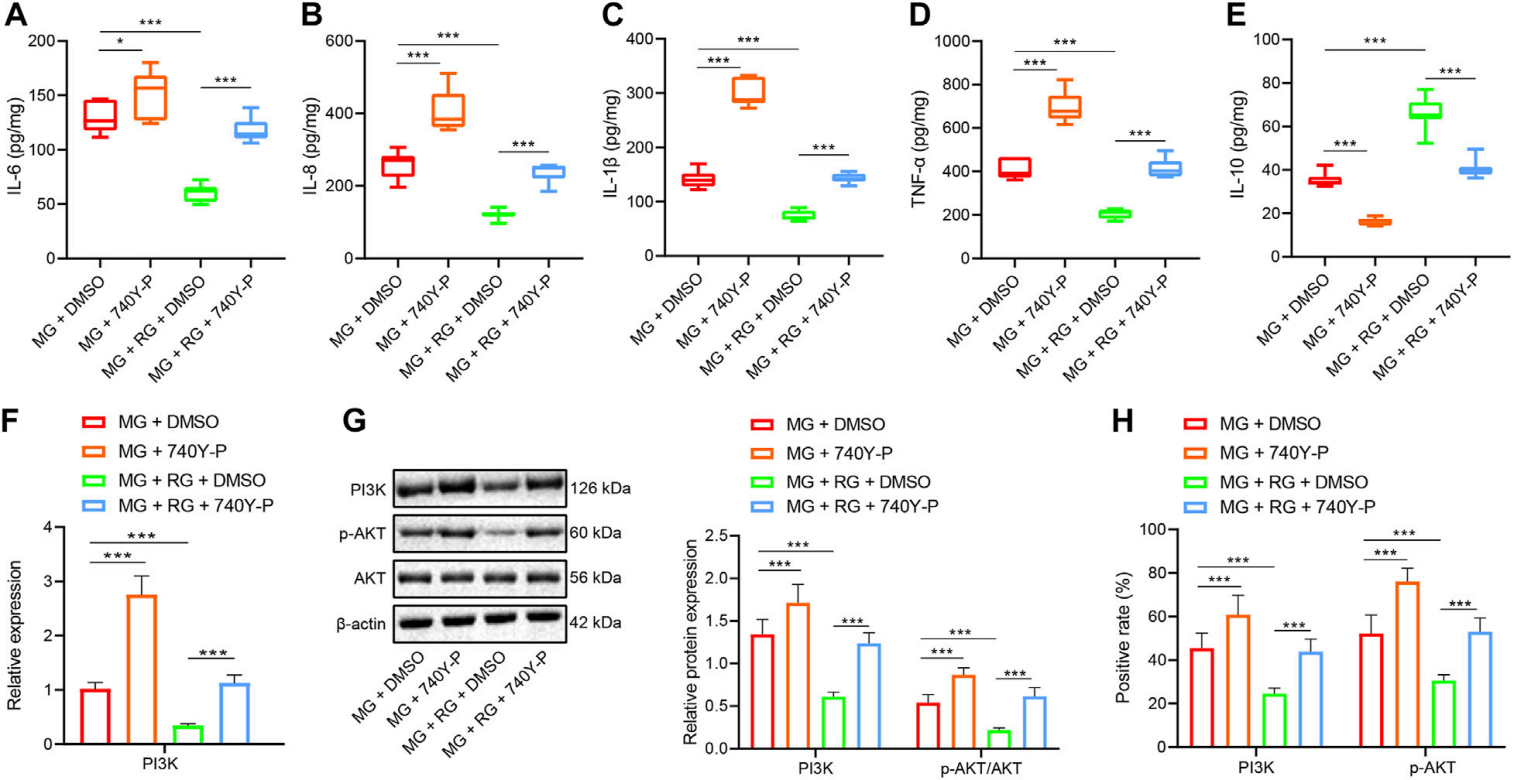
Inhibition of PI3K/Akt pathway alleviates DSS-induced UC in mice. **(A–E)** The expression of IL-6, IL-8, IL-1β, TNF-α, and IL-10 in the colon tissues of mice in different treatment groups was determined using ELISA. **(F)** The expression of PI3K in colon tissues was examined using RT-qPCR. **(G)** The expression of PI3K, p-Akt, and Akt protein in colon tissues was examined using Western blot. **(H)** The expression of VEGFA and Akt protein in colon tissues was detected using IHC. ^*^ *p* < 0.05, ^**^ *p* < 0.01, and ^***^ *p* < 0.001. *n* = 20. Measurement data were expressed by mean ± SD. One-way ANOVA was conducted for multiple group comparison, followed by Tukey’s post hoc test. MG, model control group; RG, resveratrol group; 740Y-P, PI3K/Akt activator.

The results of ELISA illustrated that administration of 740Y-P induced elevated IL-6, IL-8, IL-1β, and TNF-α expression levels (*p* < 0.01) but reduced those of IL-10 (*p* < 0.05). These findings suggested that the activation of the PI3K/Akt pathway can promote intestinal inflammation in mice. However, the administration of RSV and DMSO induced downregulated the expression levels of IL-6, IL-8, IL-1β, and TNF-α (*p* < 0.05) but augmented those of IL-10 (*p* < 0.05). These findings highlighted that RSV can reduce UC inflammation in mice. Furthermore, cotreatment of RSV and 740Y-P brought about reduced expressions of IL-6, IL-8, IL-1β, and TNF-α (*p* < 0.01) and increased IL-10 expression levels (*p* < 0.01) (Figures 4A–E). Activating the PI3K/Akt pathway can offset the therapeutic effect of RSV on intestinal inflammation in mice.

Additionally, RT-qPCR demonstrated that the treatment of 740Y-P brought about a significant elevation in PI3K expressions in mice with UC (*p* < 0.01), which could be reversed by the administration of RSV (*p* < 0.05). In the mice with UC equally treated with RSV, the administration with 740Y-P was found to induce elevated PI3K expression levels (*p* < 0.05) (Figure 4F). Meanwhile, Western blot analysis and IHC illustrated that administration of 740Y-P in mice with UC induced raised protein expression levels of PI3K and p-Akt/Akt ratio compared to those treated with DMSO (*p* < 0.01). Likewise, the administration with 740Y-P induced elevated PI3K expressions and p-Akt/Akt ratio in the mice with UC equally treated with RSV compared to those treated with DMSO (Figures 4G,H). All in all, these findings indicated that the activation of the PI3K/Akt pathway can promote DSS-induced UC in mice.

### RSV Improves the Intestinal Flora Structure of Patients With UC by Inhibiting the PI3K/Akt Pathway

**FIGURE 5:**
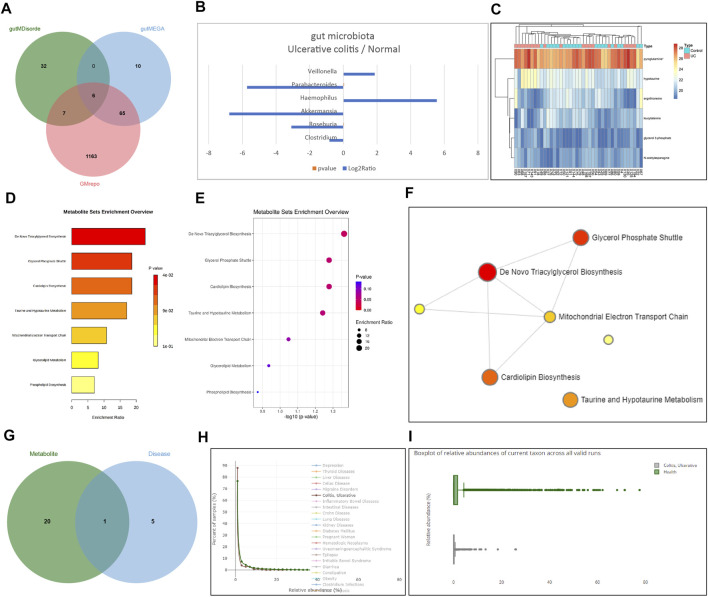
RSV improves the intestinal flora structure of UC mice by inhibiting the PI3K/Akt pathway. **(A)** The Venn diagram of the intersection of intestinal bacteria associated with UC predicted by the databases GMrepo, gutMEGA, and gutMDisorder. **(B)** Significantly different intestinal flora in the log2Ratio of UC samples and normal samples. **(C)** Differential metabolites and the expression heat map of the differential metabolites were drawn. **(D)** Bar graph of enrichment analysis results of differential metabolites. **(E)** Dot chart of enrichment analysis results of differential metabolites. **(F)** Correlation network diagram of metabolites. **(G)** The presence of differential metabolites of microbes and UC of the differential microorganisms intersects Venn map was predicted using VMH database. **(H)** Distribution map of relative abundance of *Akkermansia* in healthy samples and UC samples. **(I)** Box plot of the relative abundance of *Akkermansia* in healthy samples and UC samples.

To further clarify whether RSV could regulate intestinal symbiotic flora to restore colonic flora homeostasis, we screened a total of 1,241, 81, and 45 intestinal bacteria associated with UC using the GMrepo, gutMEGA, and gutMDisorder databases, respectively, and obtained six significantly different bacteria including *Clostridium*, *Roseburia*, *Akkermansia*, *Haemophilus*, *Parabacteroides*, and *Veillonella* by intersection (Figure 5A). In addition, the log2Ratio of the six significantly different intestinal flora in the UC sample and the normal sample was obtained through the gutMEGA database (Figure 5B). Related samples of UC were further retrieved through the Metabolomics Workbench database, and differences in metabolite expression profiles were analyzed. The following six differential metabolites were obtained: glycerol 3-phosphate, ergothioneine, N-acetylasparagine, pyroglutamine, hypotaurine, and leucylalanine, and consequently, a heat map of differential metabolite expression was plotted (Figure 5C). The enrichment analysis results of different metabolites and the correlation network diagram of metabolites were obtained through the MetaboAnalyst tool (Figures 5D–F), which displayed that metabolites were primarily involved in triacylglycerol biosynthesis, glycerol phosphate shuttle, and cardiolipin biosynthesis.

Additionally, analyses of the VMH database indicated that microbes with different metabolites were further predicted to intersect with the different microorganisms of UC to obtain *Akkermansia* (Figure 5G). We predicted that *Akkermansia* was the key microbe influencing UC through its metabolism. The distribution and box diagram of relative abundance of the key microorganism *Akkermansia* in healthy samples and UC samples were subsequently searched through the GMrepo database (Figures 5H,I), which clarified that *Akkermansia* may improve UC in mice by metabolizing the intestinal microflora structure of mice with UC. Further searching the KEGG database revealed that differential metabolites were involved in the biosynthesis of secondary metabolites (map01110). Meanwhile, RSV was also found to be implicated in this metabolic pathway, and there was an intersection of metabolites. Thus, we speculated that RSV might regulate the structure of intestinal flora by regulating related metabolites. Interestingly, a prior study has shown that *Akkermansia* has more abundant taxa in the control samples in comparison with UC samples (Shah et al., 2016), whereas RSV is also known to restore the intestinal flora such as *Akkermansia*, which is reduced due to enteritis, to a steady-state level (Alrafas et al., 2019). Altogether, these findings indicated that RSV had a tendency to restore intestinal homeostasis by regulating the structure of intestinal flora. Combined with the conclusion that RSV can improve UC by inhibiting the PI3K/Akt pathway, we concur that RSV may improve the intestinal microflora structure of UC patients by inhibiting the PI3K/Akt pathway.

## Discussion

**FIGURE 6:**
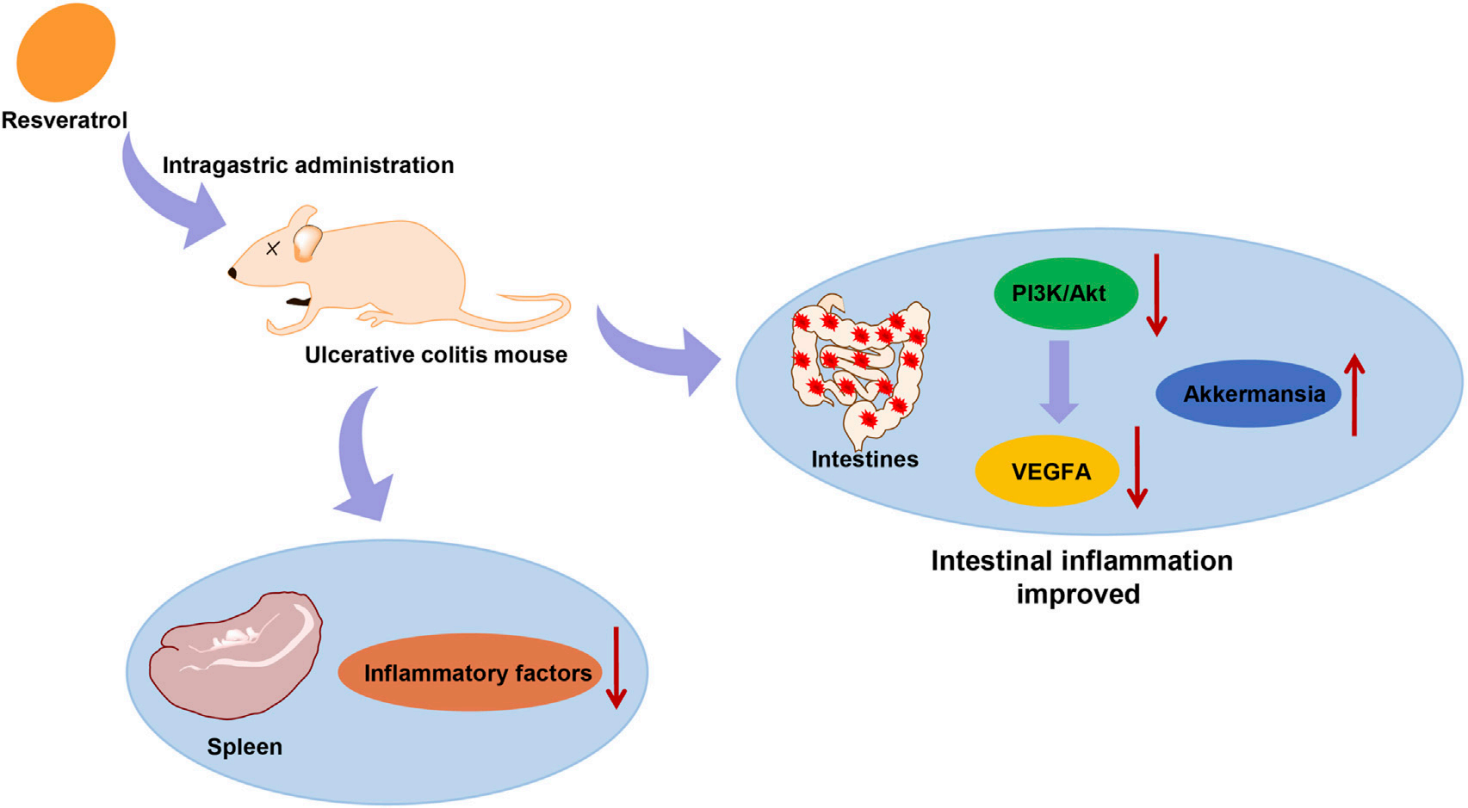
RSV suppressed the activation of the PI3K/Akt pathway and reduced the VEGFA gene expression to alleviate UC.

UC is an inflammatory disease of the bowel, whose severity changes over time (Choi et al., 2019). Evaluating the risk of UC progression and determining the best treatment plan remain a clinical challenge because the majority of patients show an incomplete response to treatment (Schirmer et al., 2018). The current study was conducted to investigate the mechanism of RSV on alleviating UC in mice by mediating intestinal microflora homeostasis through the PI3K/Akt/VEGFA pathway, and the obtained findings revealed that RSV suppressed the activation of the PI3K/Akt pathway and reduced the VEGFA gene expression to alleviate UC in mice (Figure 6).

Initial findings in our study revealed that RSV alleviated DSS-induced UC in mice. In addition, we observed that administration of RSV brought about a marked reduction in IL-6, IL-8, IL-1β, and TNF-α expressions and increased that of IL-10. Similarly, a prior study revealed that RSV treatment at a certain dose could partially improve disease activity and quality of life in patients with UC (Samsamikor et al., 2016). Meanwhile, RSV was also previously shown to exert protective effects on DSS-induced colitis in mice with UC (Liu et al., 2019). Further in line with our findings, another study illustrated that resveratrol treatment led to decreased expressions of inflammatory factors and increased expressions of tight junction proteins, thus alleviating UC intestinal mucosal barrier dysfunction (Pan et al., 2020). Furthermore, significantly elevated protein expressions of IL-1β were recently observed in inflammatory colon tissues of patients with UC, wherein the administration of RSV resulted in decreased mRNA and protein levels of IL-1β (Sabzevary-Ghahfarokhi et al., 2020). Moreover, the study performed by Yao et al*.* showed that RSV treatment significantly inhibited the severity of UC and dramatically reduced the expressions of TNF-α, IL-8, IFN-γ, p22, and gp91 relative to the UC control group, highlighting the antioxidant effects of RSV on preventing oxidative damage induced by DSS (Yao et al., 2010).  IL-10 serves as an anti-inflammatory cytokine, which is increased  after RSV administration in inflammatory bowel disease (Wang J. et al., 2020).

Subsequent experimentation in our study revealed PI3K/Akt/VEGFA as a molecular target for RSV in the treatment of UC. Interestingly, a recent report illustrated that RSV can affect the progression of intestinal injury *via* regulation of the PI3K/Akt pathway (Radwan and Karam, 2020). Expanding on their results, the current study demonstrated that the RSV could relieve the pathological condition of mice with UC by inhibiting the PI3K/Akt pathway to reduce the VEGFA gene expression *via* intestinal flora. The PI3K/Akt pathway is known to participate in the regulation and release of proinflammatory cytokines, which in turn influences the development of UC (Huang et al., 2011). In addition, correlation and interaction analysis of 22 genes on the STRING website revealed that VEGFA was the core target of the API3K/Akt pathway (Figure 1D). VEGFA was previously identified as a central target gene through the PPI network and was further known to exhibit better affinity with RSV in computer simulation studies (Wang W. et al., 2020). However, our study focuses on UC as well as the mechanism research and analysis of RSV in the intestinal flora, rather than the occurrence and development of obesity. Above all, the aforementioned findings and data indicate that RSV plays a role in UC by mediating VEGFA *via* the PI3K/Akt pathway.

Additionally, the current study illustrated that RSV inhibited the activation of the PI3K/Akt pathway to reduce VEGFA expression. The relationship between RSV and PI3K/Akt has been investigated previously; for instance, RSV was found to downregulate the protein expression of PI3K/Akt, thus attenuating the progression of intestine (Radwan and Karam, 2020). Moreover, upregulated levels of VEGFA have been documented in UC patients compared to normal controls (Pousa et al., 2011). Similarly, RSV possesses the ability to inhibit the expression of CXCR4 by reducing the phosphorylation level of NF-κB, which brings about a reduction in VEGF secretion (Seong et al., 2015). Furthermore, our findings revealed that inhibition of the PI3K/Akt pathway alleviated DSS-induced UC in mice. On the other hand, activation of the PI3K/Akt contributed to highly increased expressions of IL-6, IL-8, IL-1β, and TNF-α but reduced those of IL-10. In line with our findings, another study suggested that application of a PI3K/Akt inhibitor can improve various symptoms of UC in mice, such as reduced growth, loose stools, and stool bleeding, and further represses the activation of the PI3K/Akt pathway (Yan et al., 2020). Besides, RSV is also known to reduce TNF-α-induced IL-1β and MMP-3 production by suppressing PI3K-Akt signaling in fibroblastoid synoviocytes of rheumatoid arthritis (Tian et al., 2013). Overall, our findings indicated that RSV improved the intestinal flora structure of UC mice through inhibiting the PI3K/Akt pathway. Simultaneously, we also uncovered that *Akkermansia* may improve UC in mice by metabolizing the intestinal flora structure of UC mice. Existing literature shows that *Akkermansia* is more abundant in untreated patients with UC (Shah et al., 2016). Moreover, RSV can restore intestinal flora such as *Akkermansia* to homeostatic levels due to reduced enteritis (Alrafas et al., 2019).

Altogether, the current study revealed that RSV suppressed the activation of the PI3K/Akt pathway and reduced the VEGFA gene expression to alleviate UC in mice. Our findings may offer a theoretical basis to translate the anti-inflammatory role of RSV to clinic terms in the treatment of UC. With the help of metabolomics methods, we further discovered the alleviating effect of RSV, which may work by changing the microbiota in the intestinal tract of experimental animals. In our future endeavors, we will further evaluate the results of bioinformatics analysis and experimentally analyze the role of the intestinal microbiota from experimental animals and its relationship with RSV. In addition, experimental analysis of gut microbiota should be conducted with the help of experimental animals. Prospective studies are also expected to deal with any possible, yet unpredictable factors when using this mechanism in the clinical treatment of UC.

## Data Availability

The original contributions presented in the study are included in the article/Supplementary Material; further inquiries can be directed to the corresponding author.
