# Erratum: Resveratrol Alleviates Dextran Sulfate Sodium-Induced Acute Ulcerative Colitis in Mice by Mediating PI3K/Akt/ VEGFA Pathway

**DOI:** 10.3389/fphar.2021.797101

**Published:** 2021-11-02

**Authors:** Frontiers Production Office

**Affiliations:** Frontiers Media SA, Lausanne, Switzerland

**Keywords:** resveratrol, ulcerative colitis, phosphoinositide 3-kinase, protein kinase B, vascular endothelial growth factor A

Due to a production error, the latest version of Figure 3 was not published. The corrected Figure 3 appears below.

**FIGURE 3:**
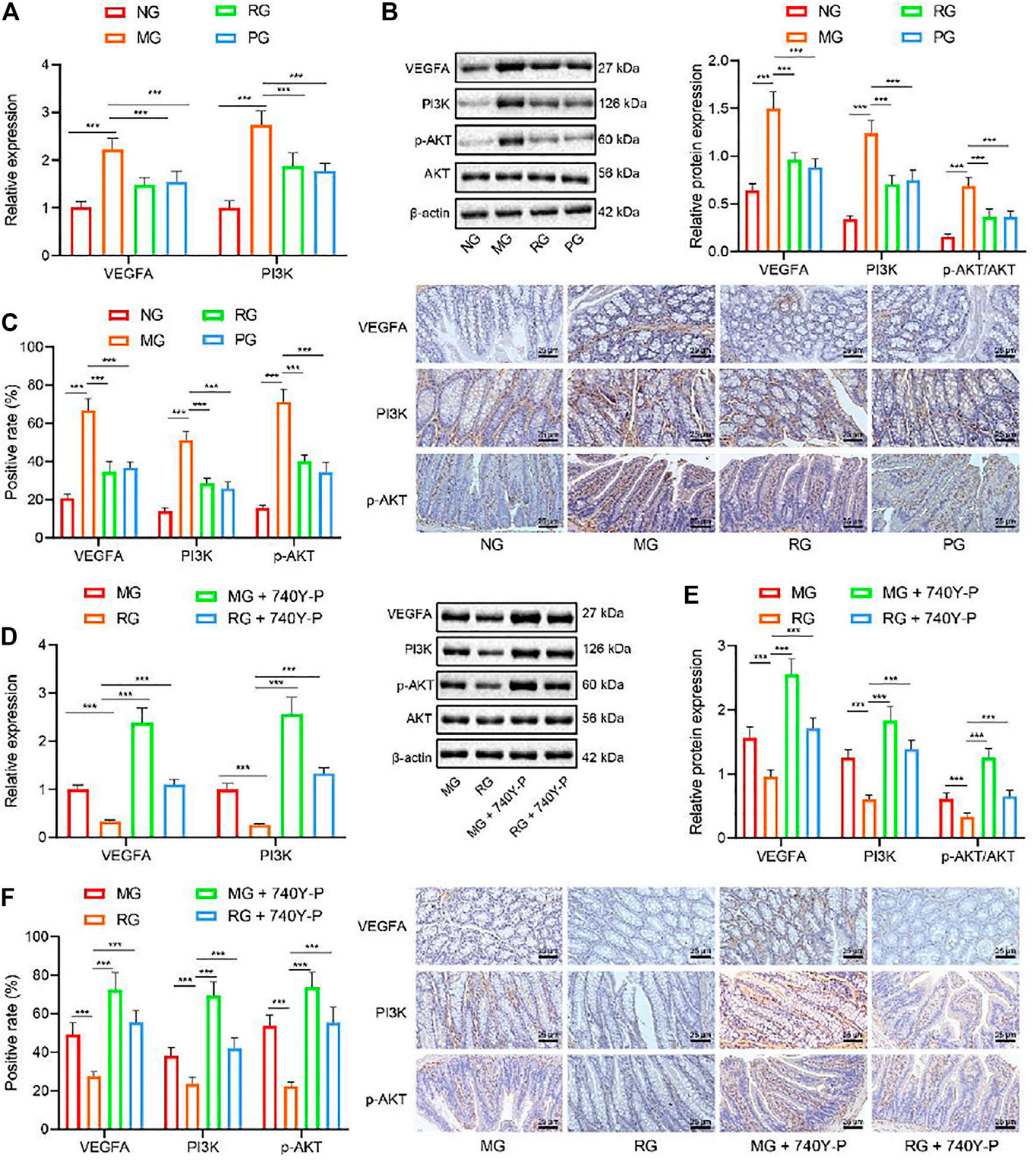
RSV inhibits the PI3K/Akt pathway activation and reduces the VEGFA gene expression. **(A)** The expression of VEGFA and PI3K and genes in colon tissues were analyzed by RT-qPCR. **(B)** The expression of VEGFA, PI3K, p-Akt, and Akt protein in colon tissues was detected by Western blot analysis. **(C)** Expression of VEGFA and Akt protein in colon tissues was analyzed by IHC. **(D)** The expression of VEGFA and PI3K in colon tissues after the addition of PI3K/Akt activator was determined with RT-qPCR. **(E)** Western blot analysis of the expression of VEGFA, PI3K, and p-Akt/Akt ratio was in colon tissues after the addition of PI3K/Akt activator. **(F)** The expression of VEGFA and p-Akt protein in colon tissue after adding PI3K/Akt activator was determined with IHC. #*p* < 0.05 vs. VEGFA, PI3K, and Akt expression in MG. ##*p* < 0.01. n 20. Measurement data were expressed by mean ± SD. One-way ANOVA was conducted for multiple group comparison, followed by Tukey’s post hoc test. NG, normal control group; MG, model control group; RG, resveratrol group; PG, positive control group; VEGFA, vascular endothelial growth factor A; 740Y-P, PI3K/Akt activator.

The publisher apologizes for this mistake. The original version of this article has been updated.

