# Supplementary material for: Resveratrol Alleviates Dextran Sulfate Sodium-Induced Acute Ulcerative Colitis in Mice by Mediating PI3K/Akt/VEGFA Pathway
Source: Front Pharmacol. 2021 Aug 23;12:693982. doi: 10.3389/fphar.2021.693982 (PMC8419259; doi:10.3389/fphar.2021.693982)
Supplement: Supplementary file 1 [file Table1.docx]

**Table S1.** Primer sequences for RT-qPCR

| Gene | Primer sequence (5’→3’) |
| --- | --- |
| PI3K | FOR: ACTTTGTGACCTTCGGCTT |
|  | REV: TCCTGTACTTCTGGATCTTTAA |
| VEGFA | FOR:  CTGTGCAGGCTGCTGTAAC |
|  | REV:  ACAGTGATTTTCTGGCTTTGTTC |
| β-Actin | FOR:  GACCTGTACGCCAACACAGT |
|  | REV:  CTCAGGAGGAGCAATGATCT |

Note: PI3K, phosphoinositide 3- kinase; AKT, protein kinase B; VEGFA, vascular endothelial growth factor-A
