# Supplementary figures and images for: Resveratrol Alleviates Dextran Sulfate Sodium-Induced Acute Ulcerative Colitis in Mice by Mediating PI3K/Akt/VEGFA Pathway
Source: Front Pharmacol. 2021 Aug 23;12:693982. doi: 10.3389/fphar.2021.693982 (PMC8419259; doi:10.3389/fphar.2021.693982)

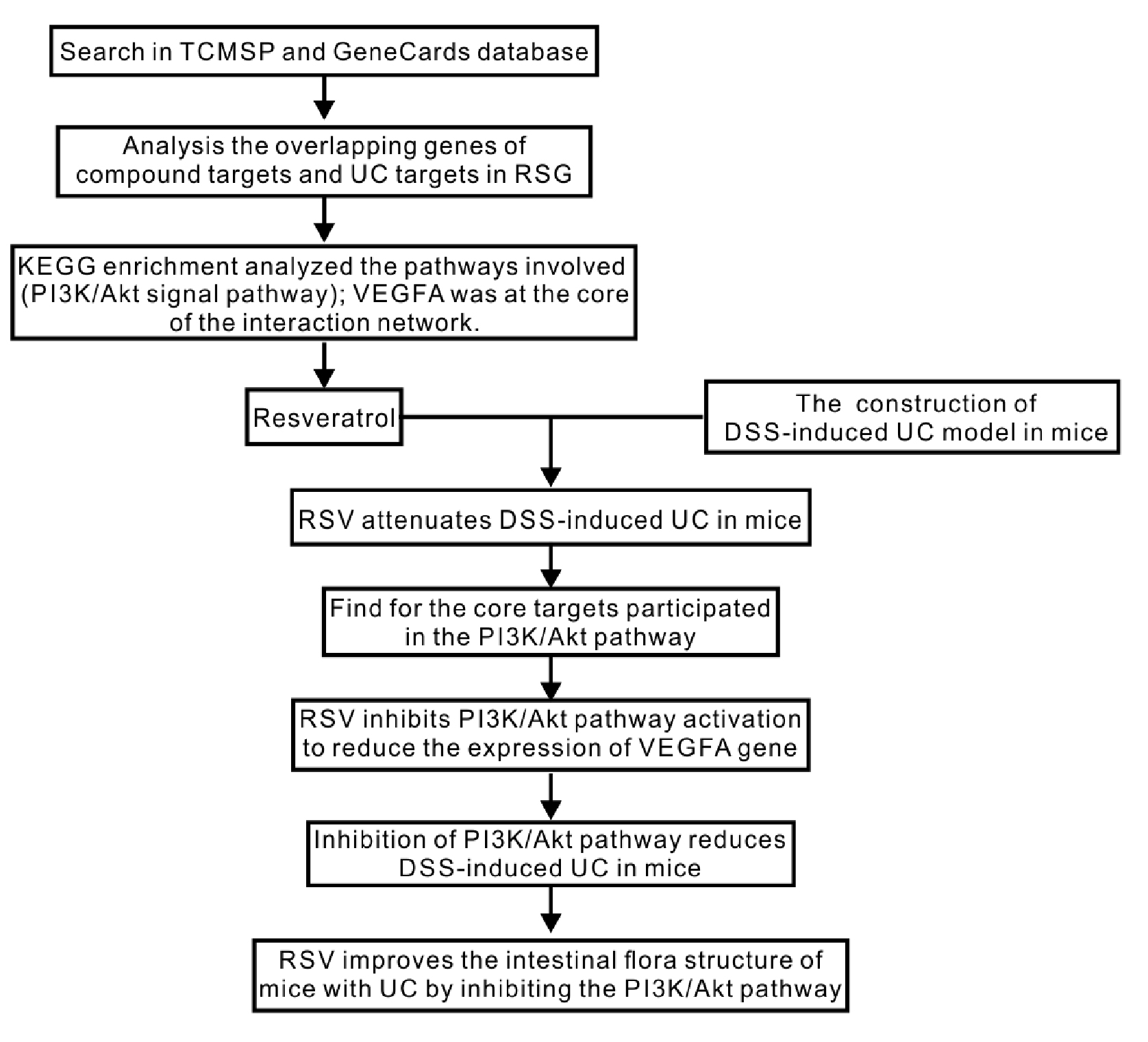

Supplement: Supplementary file 2 [file Image1.JPEG]
